# Severe Gastroparesis-like Syndrome Following Single-dose Liraglutide Administration: A Case Report: Short title: Severe Gastroparesis-like Syndrome Following Single-dose Liraglutide Administration

**DOI:** 10.31661/gmj.v15i.4135

**Published:** 2026-03-02

**Authors:** Eyad A. Makkawy

**Affiliations:** ^1^ Department of Medicine, Gastroenterology Division, Prince Mohammed bin Abdulaziz Hospital, Riyadh, Saudi Arabia

**Keywords:** Liraglutide, Gastroparesis, GLP-1 Agonist, Drug-induced Gastropathy, Refractory Vomiting, Adverse Drug Reaction

## Abstract

**Background:**

As a GLP-1 analogue, liraglutide is given to patients as a treatment for both obesity and type 2 diabetes. It taken safely most of time, though there are also reports of effects on the digestive system.

**Case Report:**

In our case, a 30-year-old healthy female developed severe slowing of her stomach after a single injection of liraglutide (1 mg) for weight loss, without a doctor’s care. Within a few hours of getting the treatment, the patient began having severe abdominal pain and kept vomiting. A thorough review using imaging and lab tests did not find anything wrong with her system to explain her symptoms. It points out that a single dose of liraglutide may result in major gastric motility problems for some patients. She recovered from her condition over two weeks with supportive care which eliminated her symptoms.

**Conclusion:**

Further study is required to explain these reactions and to set up rules for safer use of GLP-1 receptor agonists outside the diabetic population.

## Introduction

Both type 2 diabetes mellitus and obesity can now be treated with GLP-1 receptor agonists, a key treatment for both conditions [1]. They regulate the release of insulin, stop glucagon and boost feelings of fullness using different mechanisms throughout the nervous system [2]. GLP-1 agonists is known to improve metabolism, but now there is concern about how they may be affecting gastric motility and other elements of digestion [1, 3].

Many studies have looked at how GLP-1 works in controlling the movement of food out of the stomach. Endogenous GLP-1 has the effect of slowing how food passes through the stomach during neuroendocrine control of nutrient absorption [4]. Because GLP-1 receptor agonists are dosed at levels meant for treating diabetes, nausea and vomiting are common side effects [5]. At present, how these agents lead to various gastric motility disorders through brief usage is not well described in scientific literature.

Liraglutide which resembles human GLP-1 by 97%, is specially known for slowing gastric emptying [1]. Clinical trials have mostly examined the way obesity drugs affect metabolism, while cases of serious gastroparesis reported after the drugs were approved have been observed [3]. Being that a medicine for weight loss through hunger actually results in gastrointestinal problems is a serious challenge for doctors.

This new occurrence of severe and protracted gastroparesis in a patient taking liraglutide shines more light on the potential of GLP-1 agonists to cause gastric dysmotility more severely than we have realized.

## Case Presentation

A 30-year-old woman who had been well up to that point presented to the emergency room with three days of extreme pain in her stomach and ongoing vomiting. The pain stayed constant, felt dull, was only in the epigastrium without radiation and was rated at an 8 out of 10 in severity. Vomiting started after meals but grew into frequent vomiting of bile and finally prevented the patient from eating or consuming fluids at all. After self-administering one 1 mg subcutaneous dose of liraglutide for weight loss, she started having these symptoms. She denied prior such episodes, ongoing serious medical issues or regular medication use.

### Timeline

Symptoms began immediately after liraglutide injection. Vomiting progressed from postprandial to frequent bilious emesis, preventing all oral intake by day 3. Pain remained constant, dull, epigastric, non-radiating, and 8/10 in severity.

### Clinical Findings

On physical examination, the patient appeared distressed and dehydrated. Her blood pressure was 90/50 mmHg, her heart rate was between 100 and 115 beats per minute and her respiratory rate was 20 breaths per minute. During the abdominal exam, pressure in the epigastrium was tender on palpation but did not elicit guarding or rebound tenderness. The heart and lungs were normal, with no additional sounds and the neurological examination was unremarkable. Anthropometrics included weight 61 kg, height 155 cm, and BMI 25.4 kg/m².

### Diagnostic Assessment

During the laboratory investigation, abnormalities found were hypokalemia of 3.0 mmol/L, hyponatremia of 132 mmol/L and chloride 106 mmol/L. The complete blood count showed elevated white blood cells (WBC 12.0 x109/L), normal hemoglobin (13.2 g/dL) and increased platelets (390 x109/L). There was no sign of inflammation with a CRP level measuring <0.1 mg/dL. There was nothing abnormal in pancreatic enzymes (lipase 30 U/L) and the creatinine was normal at 0.53 mg/dL. Metabolic evaluation found normal blood glucose (random glucose 4.7 mmol/L) and moderate levels of ketones in the urine (++). The electrocardiogram revealed sinus tachycardia with a rate of 108 beats per minute and without evidence of ischemic changes. Tests for D-dimer were negative. Both an abdominal ultrasound and a CT of the abdomen performed externally failed to show pancreatitis, bowel obstruction or other pathology. There were no abnormalities seen on the chest X-ray. Management for refractory vomiting and dehydration was the reason for the patient’s admission. She was treated in hospital with hydration by IV, antiemetic medications including ondansetron and domperidone and proton pump inhibitor pantoprazole for gastroprotection. Because of her constant vomiting, she received nothing by mouth (NPO, nil per os) and the team decided not to place a nasogastric tube. Although she was carefully managed, oral eating triggered vomiting until hospital day 8. A gastroscopy examination done on day 7 of admission found inflammation of the stomach mucosa, most significantly in the fundus, but normal appearances in the esophagus, pylorus and duodenum. Mild reactive gastropathy was seen in the biopsies suggesting that prolonged vomiting and possible delayed gastric motility caused by the drug were factors. Therapeutic Intervention Her abdominal pain improved gradually as she remained in the hospital and by day 8 she could tolerate clear liquids. At her request, the healthcare team discharged her and initiated a liquid diet.

**Figure-1:**
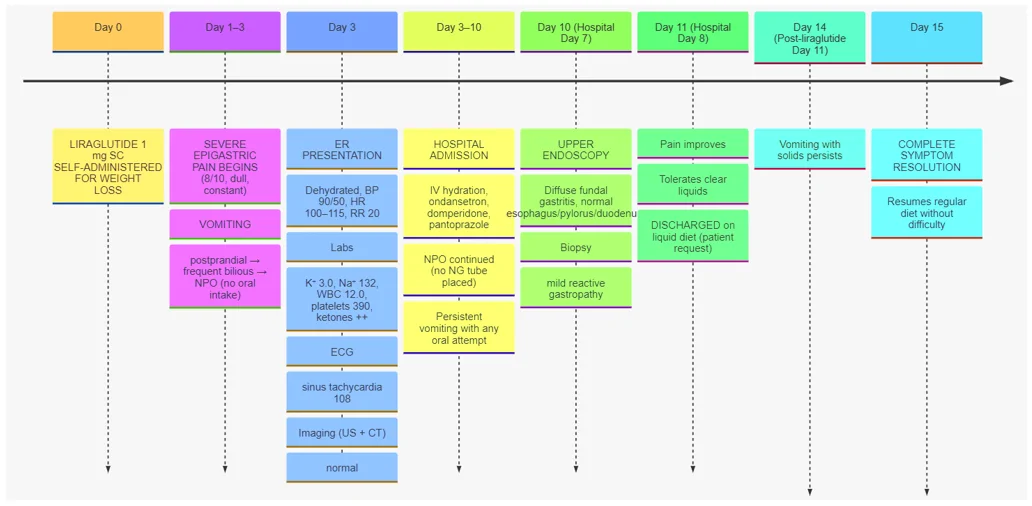
Figure 1. Timeline of Symptoms and Clinical Course After Liraglutide Administration

### Follow-up and Outcomes

Follow-up after discharge revealed she continued to experience vomiting with solid food until day 11 post-liraglutide administration. Complete resolution of symptoms occurred by day 15, at which point she was able to tolerate a regular diet without difficulty.

## Discussion

In this case, a single dose of 1 mg liraglutide of the GLP-1 receptor agonist group led to severe symptoms similar to gastroparesis. While this issue has been seen with frequent use in the past, the same severe and fast impacts occur after just one dose. Refractory vomiting, unable to tolerate gastric content and endoscopic data of gastropathy with no mechanical obstruction are consistent with recent GLP-1 agonist studies on gastroenterology, but they also expand the known range of the condition.

Several investigations have discovered that using GLP-1 agonists often results in gastric emptying being slowed, generally only at higher doses. Horowitz et al. [6] found that liraglutide increases the time it takes gastric contents to leave the stomach in people with type 2 diabetes, an action due to its effects on muscle and nerves in the stomach. Recent findings in our patient show that just one overdose of loperamide can cause serious gastrointestinal dysfunction in people who are at risk. That result is consistent with a case report [7] showing acute gastroparesis in three patients after starting liraglutide, but all health conditions resolved within 72 hours after stopping the drug. For fifteen days, our patient continued to show symptoms, suggesting that this was a worse and longer-lasting form of the phenomenon.

Our patient’s clinical presentation matches the histopathology we see from their endoscopy. The results from a prior study [8] were similar to what was seen in our patients. Biopsies from those patients displayed nonspecific inflammation and edema. It was believed by the authors that mucosal irritation occurs as a result of delayed transfer of food and its acid into the stomach.

This hypothesis is supported by our observations, as the patient got better when gastric motility was back to normal.

Symptoms seen in our patient are both more serious and last longer than the average symptoms described in clinical trials. The LEADER trial [9] which checked liraglutide’s cardiovascular effects, recorded nausea and vomiting in nearly one-third of participants with no cases of lasting gastroparesis. This difference may be explained by trials not spotting such problems or because some patients are particularly vulnerable. Work by Davies and colleagues [10] revealed that changes in GLP-1 receptor genes might cause a strong response in the intestines in some individuals when they consume a standard dose of the peptide.

Supportive care is still considered the best treatment for this condition, as is shown by the patient’s benefits from IVs and antiemetics. Our results with prokinetics are similar to what Lembo et al. [11] saw, as traditional gastroparesis therapy frequently does not help in drug-induced cases. This suggests that changes in movement caused by GLP-1 agonists may require new treatment strategies because the body responds differently to them.

Our case contrasts sharply with the overdose scenario reported by Bin Nafisah et al. [12], where a 28-year-old diabetic patient inadvertently injected 18 mg and developed acute epigastric pain radiating to the back, persistent vomiting, and relative hypoglycemia (4.6 mmol/L) requiring multiple dextrose boluses over 16 hours of observation. While both patients presented with prominent gastrointestinal distress, epigastric tenderness and bilious emesis, our case demonstrated prolonged gastric stasis lasting 15 days without glycemic perturbation (random glucose 4.7 mmol/L, urinary ketones ++ from starvation), whereas the overdose induced transient metabolic instability resolved within hours. This temporal dissociation suggests a dose-threshold effect: massive overdose triggers rapid pancreatic β-cell overstimulation and insulin surge, whereas therapeutic-range exposure in a liraglutide-naïve individual may predominantly amplify delayed gastric emptying via vagal inhibition and direct smooth-muscle GLP-1 receptor effects, unmasking gastroparesis without hypoglycemia.

Endoscopic findings in our patient including the diffuse fundal gastritis with reactive gastropathy, diverge from the normal pancreatic enzymes and absent structural pathology in both the overdose case and the liver-injury cohort described by Lai et al. [13] In Lai’s 30-year-old female, liraglutide (uptitrated to 1.8 mg daily over 19 weeks) precipitated hepatocellular injury (peak ALT 1,098 IU/L, R-ratio 32.8) with eosinophilic portal inflammation, resolving only after 6 months off-drug, whereas our patient’s gastric mucosal changes emerged acutely and remitted fully by day 15 post-exposure. The contrasting organ tropism, stomach versus liver, may reflect individual differences in GLP-1 receptor density or downstream signaling; gastric chief cells express high GLP-1R levels responsive to even single-dose pharmacodynamics, whereas hepatocyte injury likely requires cumulative oxidative stress or idiosyncratic immune activation. Notably, neither case exhibited pancreatitis despite liraglutide’s known risk, reinforcing that gastric dysmotility and hepatotoxicity represent distinct, non-overlapping adverse pathways.

When viewed alongside therapeutic-success narratives, our case shows liraglutide’s paradoxical profile. Ashraf et al. [14] documented sustained 20-lb weight loss over 6 months in hypothalamic obesity using 3 mg daily, with restored satiety and no gastrointestinal intolerance, while Caputo et al. [15] achieved 13.3% body-weight reduction in Beckwith-Wiedemann syndrome over 20 months at the same dose.

Both reports show enhanced GLP-1 signaling correcting central hunger dysregulation, yet our patient, lacking hypothalamic lesions, experienced amplified peripheral gastric inhibition from a single exposure, suggesting GLP-1R hypersensitivity in the enteric nervous system. The rapid onset (within hours) and complete resolution upon drug clearance imply reversible pharmacodynamic exaggeration rather than structural damage, unlike the protracted hepatotoxicity requiring months for enzymatic normalization.

## Conclusion

The study showed that using liraglutide can lead to a serious, lingering form of gastroparesis, even in a normally healthy patient. Clinicians should keep an eye out for this chance, primarily in patients who vomit soon after starting GLP-1 agonists.

## Conflict of Interest

The authors declare no conflict of interest.

## AI Disclosure Statement

During the preparation of this manuscript, the authors used ChatGPT, OpenAI company for language editing, grammar improvement, and liboberry.com for reference management. After its use, the authors thoroughly reviewed, verified, and revised all AI-assisted content to ensure accuracy and originality. The authors take full responsibility for the integrity and final content of the published article.
